# Community health worker knowledge and management of pre-eclampsia in southern Mozambique

**DOI:** 10.1186/s12978-016-0220-2

**Published:** 2016-09-30

**Authors:** Helena Boene, Marianne Vidler, Orvalho Augusto, Mohsin Sidat, Eusébio Macete, Clara Menéndez, Diane Sawchuck, Rahat Qureshi, Peter von Dadelszen, Khátia Munguambe, Esperança Sevene, the CLIP Feasibility Working Group

**Affiliations:** 1 Centro de Investigação em Saúde da Manhiça (CISM), Rua 12, Vila da Manhiça, CP 1929 Moçambique; 2 Department of Obstetrics and Gynaecology, and the Child and Family Research Institute, University of British Columbia, 950 West 28th Avenue, Vancouver, V5Z4H4 Canada; 3 Universidade Eduardo Mondlane, Faculdade de Medicina, Av. Salvador Allende, 702 R/C, Maputo, Moçambique; 4 Ministério da Saúde, Av. Eduardo Mondlane, Maputo, 1008 Moçambique; 5 Barcelona Institute for Global Health (ISGlobal) /Hospital Clinic - Universitat de Barcelona, Calle Rosselló, 132, Barcelona, 08036 Spain; 6 Department of Research, Vancouver Island Health Authority, Victoria, V8R 1J8 Canada; 7 Division of Women and Child Health, Aga Khan University, Karachi, Pakistan; 8 Department of Obstetrics and Gynaecology, St George’s University London, London, SW17 0RE UK

**Keywords:** Community health workers, Knowledge, Pre-eclampsia, Eclampsia management, Mozambique

## Abstract

**Background:**

Mozambique has drastically improved an array of health indicators in recent years, including maternal mortality rates which decreased 63 % from 1990–2013 but the rates still high. Pre-eclampsia and eclampsia constitute the third major cause of maternal death in the country. Women in rural areas, with limited access to health facilities are at greatest risk. This study aimed to assess the current state of knowledge and the regular practices regarding pre-eclampsia and eclampsia by community health workers in southern Mozambique.

**Methods:**

This mixed methods study was conducted from 2013 to 2014, in Maputo and Gaza Provinces, southern Mozambique. Self-administered questionnaires, in-depth interviews and focus group discussions were conducted with CHWs, district medical officers, community health workers’ supervisors, Gynaecologists-Obstetricians and matrons. Quantitative data were entered into a database written in REDCap and subsequently analyzed using Stata 13. Qualitative data was imported into NVivo10 for thematic analysis.

**Results:**

Ninety-three percent of CHW had some awareness of pregnancy complications. Forty-one percent were able to describe the signs and symptoms of hypertension. In cases of eclampsia, CHWs reported to immediately refer the women. The vast majority of the CHWs surveyed reported that they could neither measure blood pressure nor proteinuria (90 %). Fewer reported confidence in providing oral antihypertensives (14 %) or injections in pregnancy (5 %). The other community health care providers are matrons. They do not formally offer health services, but assists pregnant women in case of an emergency. Regarding pre-eclampsia and eclampsia, matrons were unable to recognise these biomedical terms.

**Conclusions:**

Although CHWs are aware of pregnancy complications, they hold limited knowledge specific to pre-eclampsia and eclampsia. There is a need to promote studies to evaluate the impact of enhancing their training to include additional content related to the identification and management of pre-eclampsia and eclampsia.

**Electronic supplementary material:**

The online version of this article (doi:10.1186/s12978-016-0220-2) contains supplementary material, which is available to authorized users.

## Plain English summary

Maternal mortality is an important public health issue in Mozambique despite the fact that some of these deaths are related to avoidable conditions. Women in rural areas, with limited access to health facilities are the most affected. Hypertensive disorders of pregnancy (HDP) are the third major cause of maternal death in the country. This study aimed to assess the current state of knowledge and routine practices regarding HDP by community health workers (CHW). This mixed methods study was conducted from 2013 to 2014, in southern Mozambique involving CHWs, district medical officers, community health workers’ supervisors, Gynaecologists-Obstetricians and matrons. Ninety-three percent of CHW had some awareness of pregnancy complications and forty-one percent were able to describe the signs and symptoms of hypertension. In case of severity, CHWs reported to immediately refer the women. The vast majority of the CHWs surveyed reported that they could neither measure blood pressure nor proteinuria (90 %). Fewer reported confidence in providing oral antihypertensives (14 %) or injections in pregnancy (5 %). Although CHWs are aware of pregnancy complications, they hold limited knowledge specific to HDP. There is a need to promote studies to evaluate the impact of enhancing their training to include additional content related to the identification and management of HDP.

## Background

Mozambique has progressively improved an array of health indicators over the last two decades, including a 63 % decrease in maternal mortality from 1,300 in 1990 to 480 deaths per 100,000 live births in 2013 [1]. However, women still have a 1 in 40 lifetime risk of maternal death [2] chiefly due to avoidable causes including post-partum hemorrhage, maternal sepsis and pre-eclampsia and eclampsia that still need to be addressed [3, 4]. The hypertensive disorders of pregnancy (HDP) contribute significantly to high rates of maternal and perinatal death. In Mozambique, eclampsia alone is the third most common obstetric cause of maternal death [5]. The shortage of health professionals capable of responding to the need to reduce maternal mortality is also a concern. In 2011, the total number of medical doctors in the National Health System in Mozambique was 1,268 for a population of approximately 22.3 million people (5.6 medical doctors per 100,000 inhabitants) [6], putting Mozambique in position of one of the worse countries according to the World Health Organization (WHO) Work Force Observatory [7]. To overcome the shortage of medical doctors in the country, nurses and clinical officers have been trained to take some of their duties [8].

To best reach vulnerable population in light of the 1978 Alma Ata Declaration, calling for primary health care for all at a time of widespread health care worker shortages in low and middle-income countries (LMIC) [9], many countries with lack of human resources adopted polices to train non-physician clinicians mainly to support care of women and children [10]. A reflection of such policies was the development of community health worker (CHW) programmes to expand access to maternal and child care particularly in rural areas [11–13].

Mozambique introduced CHWs, known as *Agentes Polivalentes Elementares* (APE), in 1978. CHWs are selected from and serve the communities in which they live, with a high level community participation in their selection process. CHWs are expected to dedicate 80 % of their time on activities related to health promotion and diseases prevention and 20 % to provide basic curative care [14]. Some of their activities related to maternal care include promotion of antenatal care and post-partum visits, promotion of health facility based deliveries and exclusive breast feeding, identification of warning signs in pregnancy and referral to the health facility. Regarding child care apart of health promotion and some disease prevention knowledge, they are trained to manage malaria, diarrhoea and upper respiratory tract infections, identify warning signs in the new born and refer [14]. The initial CHW training lasts 14–18 weeks and additional refresher courses are provided regularly [14]. Particularly to pre-eclampsia and eclampsia nothing was included in their training manual.

Pre-eclampsia is a particularly complex condition. Only recently non-physician clinicians within the health system are being trained to manage this disease. A study in Malawi demonstrated that different types of non-physician clinicians are capable to care for pregnant women including identifying and providing preliminary care before referring to nearest health facility for further care [15]. In 2012, the WHO report on optimization of health workers’ roles, recommended that a variety of health care providers within the health system level should be involved in the care of women with pre-eclampsia and eclampsia (namely nurses, midwives and associate clinicians) by providing antihypertensive and magnesium sulphate (MgSO_4_) when appropriate [16]. Specifically for CHWs, there is lack of evidence regarding their potential role in the management of pre-eclampsia and eclampsia. Often CHWs are seen as option for care mainly in rural areas which leads to progressive increase in their scope of work and tasks. Recently, in Mozambique, for example, CHW were trained to also provide injectable contraception [17]. There are certain concerns regarding the ability of CHWs providing more differentiated health services, particularly due to their limited literacy and numeracy level and also because of their already relatively high workload. However, being present in rural communities with limited or no access to health care services, CHWs are regarded as most available option. Thus, within this scenario, we asked whether CHWs could be involved in the management of pre-eclampsia and eclampsia and thus contribute for further reduction in maternal mortality given that this condition is the third leading cause of maternal mortality.

The aim of this article is to present the current state of knowledge and practices among CHWs regarding pre-eclampsia and eclampsia in southern Mozambique. A better understanding of CHWs perspectives regarding pre-eclampsia and eclampsia management could also be useful for policy makers to design effective training strategies to improve maternal health care. Furthermore, the paper also aimed to identify gaps in current knowledge and guide future studies related to CHWs involvement in innovative packages of care such as to control and mitigate effects of pre-eclampsia and eclampsia in remote and rural communities.

For the purpose of this manuscript, CHWs are represented by formal community agents locally named “Agentes Polivalentes Elementares – APEs”. Further, because at community level, pregnant women are also frequently cared by matrons, who are elder women in the community experienced in traditional methods of care whose skills are acquired on the basis of experience and usually taught by older and more experienced matrons [18, 19], they were also a subject-matter of this study.

## Methods

### Study area

**Fig. 1:**
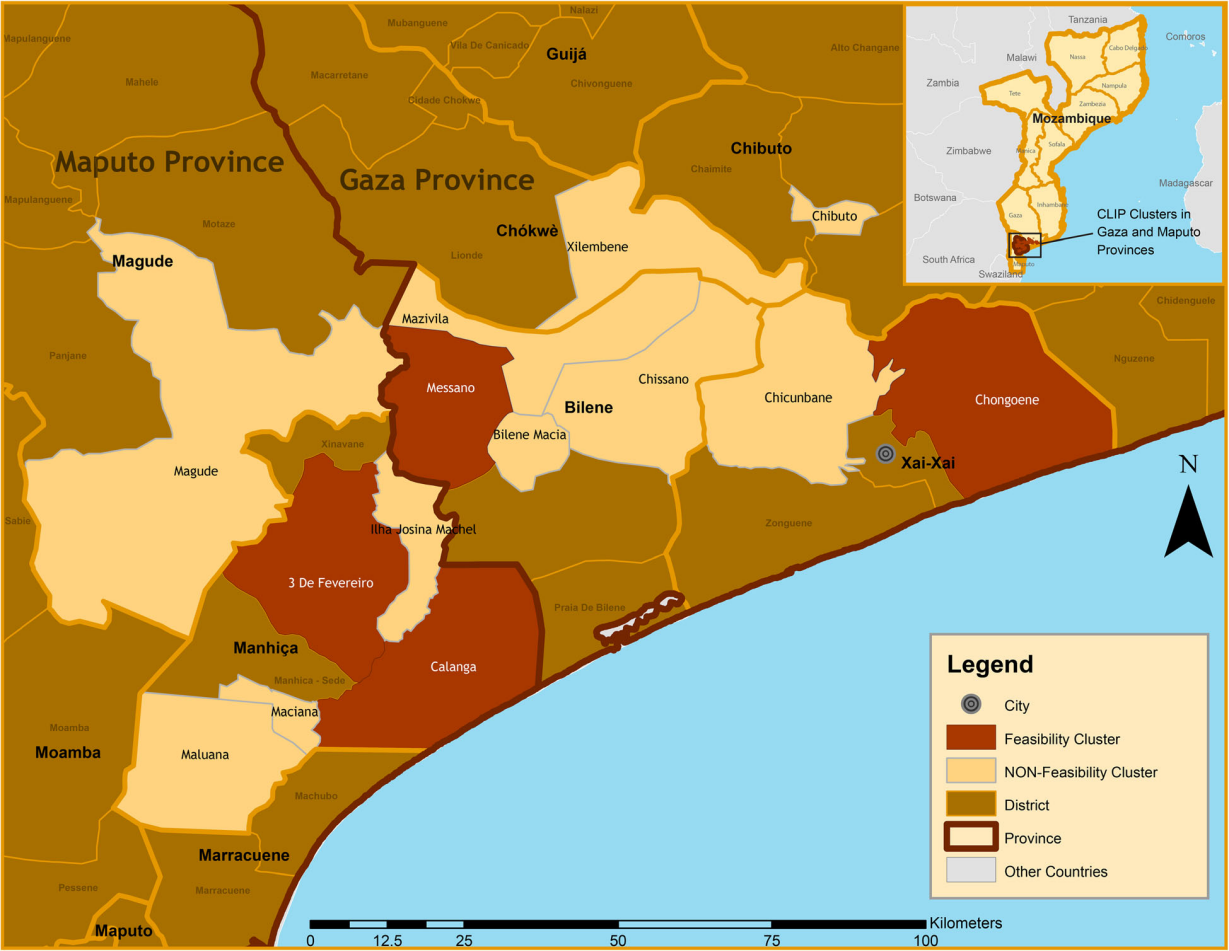
Map of the study area

This is a component of multi-country national cluster randomized control trial (cRCT) implemented in Nigeria, Mozambique, Pakistan and India, the CLIP (Community Level Interventions for Pre-eclampsia) study (NCT01911494). In Mozambique the cRCT is implemented in two provinces, namely Gaza and Maputo, in the southern part of the country (Fig. 1). This study aims to evaluate a community based intervention consisting in measure of blood pressure and proteinuria, clinical management of severe pre-eclampsia with metildopa and magnesium sulphate, ability to timely refer women to the nearest health facility and thus contribute for the reduction of maternal morbidity and mortality due to pre-eclampsia and eclampsia.

Southern Mozambique is geographically diverse, with coastal regions as well as large areas of landlocked agricultural land. Maputo Province includes the capital city, Matola, located 10Km west of Maputo city, the country’s capital. Maputo province has a population of 1.098 million. Gaza Province has a total population of 1.362 million [20]. In general, provinces are divided into districts, administrative posts (AP), localities and neighbourhoods. Each AP covers roughly 500–2,000 inhabitants. The APs included in this study area (Calanga, Maluana, Ilha Josina Machel, Três de Fevereiro, Magude, Messano, Macia, Xilembene, Chissano, Mazivila, Chongoene, Chicumbane and Chibuto) are largely impoverished rural areas where the predominant occupations are agriculture, livestock rearing, informal trading, migrant labour (mainly to South Africa), handicrafts, and work in private sugar and rice processing farms. Residents of these APs are mostly of the Changana ethnic group and speak a local dialect of the same name (for more details see Table 1).

**Table 1:** Study site characteristics

Province	Regions	Population	Population of women of reproductive age	Number of localities	Number of health facilities	Number of existing CHWs	Number of CHWs interviewed
Maputo	Maluana-Maciane	13,599	2,481	3	2	4	4
	Ilha Josina Machel-Calanga	5,720	935	4	2	8	8
	Três de Fevereiro	25,359	4,089	3	4	3	3
	Magude	27,388	3,662	7	7	27	23
Gaza	Messano	9,862	1,671	3	2	3	3
	Macia	22,349	3,248	2	1	4	4
	Xilembene	19,501	2,933	1	1	5	5
	Chissano	18,286	2,950	3	3	3	3
	Mazivila	14,875	2,215	3	2	4	4
	Chicumbane	15,684	2,385	1	3	9	9
	Chibuto	192,927	55,382	19	15	17	9
	Chongoene	19,501	2,933	6	6	6	6
Total	12	385,051	84,884	55	48	93	81

Source: Unpublished data from demographic surveillance, health facility assessment (2014) and INE (2007)

### Study design

This study is based on a formative research exercise conducted in preparation to the CLIP trial. The formative research comprised a mixed methods design, a detailed description of these methods is presented elsewhere [21].

Data collections was conducted based on forms and guides, which were developed centrally by the study coordination team, used in the other countries where the study had been previously conducted (Nigeria, India and Pakistan), and adapted to the local context of Mozambique.

Quantitative data were collected through self-administered questionnaires completed by CHWs. The qualitative data were obtained through focus group discussions with matrons, and in-depth interviews with CHWs supervisors, district chief medical officers and Gynaecologists-Obstetricians. While CHWs, CHW supervisors, and district medical officers from all study area were eligible to participate, matrons were drawn from selected AP, namely Ilha Josina Machel-Calanga, Três de Fevereiro, Messano and Chongoene.

Data collection was complemented by a desk review of existing documents regarding involvement of CHWs in maternal and child health such as policies, guidelines, reports and manuals.

Self-administered questionnaires targeted all active CHWs within the study. Recruitment was done through contacts with the health facility to which they are linked. Data collection was conducted either in the health facility where each CHW reports or at the house of the CHW.

Focus groups were conducted with matrons. This group was selected because it is also considered as community based alternative point of care for pregnant women and it was important to explore their views and practices regarding pregnancy complications (including high blood pressure and convulsions), pregnancy management (antenatal care and treatment provision) to gain an understanding of the role of matrons in the context of expansion of the maternal health care at community level. As matrons are not formally linked to the health facility and are not formally organized as a group therefore there is no clear ways to systematically identify and track them, their total number in the study area is unknown. After being identified with the assistance of neighbourhood chiefs they were invited to participate in the study. Focus groups were conducted either at the *círculos* (the usual community gathering location), or at the community leaders’ house, as groups could easily be convened in these locations.

In-depth interviews, which involved all CHW supervisors and district medical officers from the study area were conducted one-on one in the work place of the respondents. The entry points were the district level medical officers themselves who in turn identified the CHWs supervisors. As there are no Gynaecologists-Obstetricians in none of the selected districts, they were identified through *the Associação Moçambicana de Ginecologia (AMOG)* – The Mozambican Gynaecologists and Obstetricians Association.

### Study procedures

#### Desk review

It was conducted to obtain information about existing CHWs and their distribution among study sites, their training profile and scope of work. A variety of documents were reviewed, and both published and unpublished information accounted for this exercise. Most of the published documents were downloaded from the Mozambique Government portal. These included formal policy documents and other official documents such as community involvement strategies, CHW training programmes, monitoring and evaluation manuals, and meetings’ minutes and reports.

#### Data collection

Data were collected between October 2013 and May 2014. Questionnaires were designed to obtain information concerning CHW preparedness, knowledge and reported skills to manage pregnant women and to perform home-based basic treatment for women with pre-eclampsia. For the purpose of this study we assessed the following warning signs: high blood pressure, hemorrhage and convulsions. The questionnaire included 33 items on a five-point Likert scale. This format was regarded as appropriate to assess CHWs knowledge, attitudes and practices of CHW and level of confidence regarding maternal health care provision, and compare findings not only among all CHWs’ within the study area, but also eventually across the countries involved in the study. In addition, one open-ended question for respondents’ comments or additional information was included. Depending on the number of CHWs per community, individual or collective briefing sessions were held to provide instructions on how to fill it, and when required further clarification was given in Changana. Five trained local social science research assistants were available for clarification when required. The questionnaire took on average 20 min to be completed by participants.

Focus groups discussion were used to explore the views of matrons regarding pregnancy complications (including high blood pressure and convulsions), pregnancy management (antenatal care and treatment seeking), and existing health care delivery practices. Based on the FGD guide, earlier mentioned the same trained local social science research assistants facilitated the discussions, which took on average 60 min and were audio recorded.

In-depth interviews were conducted with Gynaecologists-Obstetricians, district medical officers and CHW supervisors to allow further probing on pertinent issues, such as their opinions regarding CHW’ ability to identify warning signs in pregnancy, manage pregnancy complications, and their proficiency to administer medications. These interviews were conducted by two social scientists. Interviews lasted between 30–60 min and were conducted in the workplace of participants. Field notes and audio recordings were taken at the time of in-depth interviews.

All data collection was led by a Mozambican social scientist assisted by 5 social science research assistants employed by CISM. These researchers were selected due to their familiarity with the local socio-cultural context, the research topic and their relevant qualitative and quantitative data collection expertise. Team members were fluent in Portuguese and Changana, included both male and female, and had no prior relationship with the participants. The data collection and analyse strategy was overseen by the study PI and co-PI.

#### Data management and analysis

Information obtained through the desk review was systematized and summarized to extract relevant information regarding CHWs history, role and challenges with regards to maternal and child health care that was already part of the scope of work of CHWs.

All data captured through questionnaires were sent to the Manhiça Health Research Center (CISM) for data entry and management using REDCap [22]. Double data entry was completed in all questionnaires. The presence of social science research assistants during the self-administration of the CHWs questionnaires helped to maximise the data integrity. Before it was sent to the data Center, the study team members made a revision of each questionnaire while in the field. The failures to validation rules and double data entry discrepancies were checked through queries that led to confrontation with the paper forms. Outliers and missing values were also checked. Data was then exported to Stata 13 (Stata Corp., College Station, Texas, USA) for further statistical analysis. The demographic characteristics of CHWs and the study variables of interest are presented using descriptive statistics (absolute and relative frequencies, ranges, averages and quartiles). The exact logistic regression based odds-ratio and its 95 % confidence intervals were used to describe the association between the self-reported ability of the CHWs to recognise warning signs in pregnancy with their demographic characteristics (age, sex, education and years of experience). Given the sample size no multivariate analysis was attempted.

Focus group discussions and in-depth interviews were digitally recorded using Olympus AS-2400 PC® recorders. Together with the open-ended question from CHWs questionnaire they were transcribed *verbatim* by the same team members who conducted data collection. While in-depth interviews data and that from the open-ended question of the CHW self-administered questionnaire were collected and transcribed in Portuguese. The focus group discussions were held in Changana and translated to Portuguese while being transcribed. Quality control of transcripts was ensured by listening to audio recordings and comparing them against the transcripts to confirm accuracy.

**Fig. 2:**
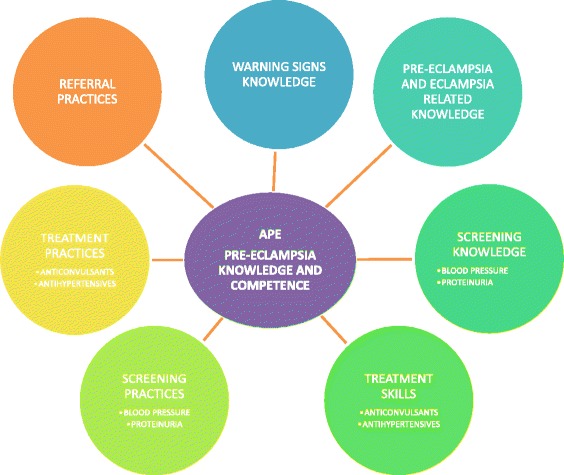
Theme structure

The qualitative data were analysed using NVivo version 10.0 (QSR International Pty. Ltd. 2012). A thematic analysis approach was taken (see Fig. 2). The coding structure (based on free nodes, branched nodes, attributes and some pre-determined queries) was developed in advance based on the study objectives through a collaborative discussion between researchers at CISM and University of British Columbia (UBC). Themes were subsequently adjusted and new themes added as they emerged from the data. As analysis was to be performed by two teams (CISM and UBC), the coding structured was in English.

The two Mozambican social scientists coded all transcriptions in Portuguese, by reading the text in Portuguese and labeling the concepts using the codes which were written in English. Three IDI and two FGD transcriptions were translated from Portuguese to English and coded by a social scientist based at UBC for three purposes: first, to support the discussions on the development of the coding structure; second, for the UBC collaborator to be familiar with the raw data, so as to assist interpretation; and finally, for quality control of the coding.

To allow the two teams to work independently, the data was split into two Nvivo projects, but the same coding structure was used for both teams. Coding consensus meetings to discussing data analysis strategy and findings were held via Skype™. Coding agreement between the coders was very high. When the coding was completed the analysed data was merged into single project managed by the Mozambican team, form which the final queries were run.

### Ethical considerations

Ethical approval for this study was granted by the CISM Institutional Review Board in Mozambique (CIBS_CISM/08/2013), as well as by the UBC C&W Research Ethics Board in Canada (H12-00132). Written informed consent was sought from each participant before data collection. For the illiterate participants a literate witness was involved in the consent process whereby they were asked to read and explain to the participant the contents of the participant information sheet. The consent form was signed by the witness and the field worker, after the participant finger print was taken. All identifiable data of participants were codified through attribution of unique identification numbers or pseudonymous to guarantee anonymity. When needed the respondent was identified by stating the administrative post or the province in the illustrative quotes.

## Results

### Participant characteristics

In total, 81 CHW were recruited to the study; corresponding to 87 % of all CHW in the study area (see Tables 1 and 2). Four CHW, all from Magude, were not included because they were not reachable at the time of recruitment to this study. Eight CHWs, all from Malehice, were not included because data collection took place around the flooding period when access to and communication with the health facilities, the recruitment entry points and data collection locations was not possible. All eighty-one questionnaire respondents were employed as CHWs at the time of data collection. Of those, 65 % were female. For more details on participants’ characteristics see Table 2.

**Table 2:** Questionnaire and in-depth interview Participants’ demographic information

Characteristic	CHWs (%) *N* = 81	Other health professionals (%)^a^ *N* = 8
Age		
20-29	14 (17 %)	1 (12 %)
30-39	12 (15 %)	3 (38 %)
40-49	25 (31 %)	3 (38 %)
> 50	21 (26 %)	1 (12 %)
Missing	9 (11 %)	0(0 %)
Gender		
Male	20 (25 %)	3 (38 %)
Female	53 (65 %)	5 (62 %)
Missing	8 (10 %)	0(0 %)
Marital status		
Married	26 (32 %)	3 (38 %)
Divorced	3 (4 %)	0(0 %)
Widowed	5 (6 %)	0(0 %)
Single	38 (47 %)	5 (62 %)
Missing	9 (11 %)	
Highest level of education attained		
Primary level uncompleted	39 (48 %)	0(0 %)
Primary level completed	15 (19 %)	0(0 %)
Secondary level uncompleted	14 (17 %)	0(0 %)
Secondary level completed	1 (1 %)	4 (50 %)
Higher degree	0 (0 %)	4 (50 %)
Missing	12 (15 %)	
Years of experience has CHW		
one year year	21 (26 %)	NA
Two years	15 (19 %)	NA
Three years	6 (7 %)	NA
˃3 years	29 (36 %)	NA
Missing	10 (12 %)	NA

^a^3 CHW supervisors (Bilene-Macia, Manhiça, Xai-Xai), 3 district medical officers (Bilene-Macia, Manhiça, Xai-Xai), 2 Gynaecologists-Obstetricians (Maputo city)

Focus groups discussions were carried out with matrons from Ilha Josina Machel-Calanga, Três de Fevereiro, Messano and Chongoene, one for each AP. A total of five focus groups were conducted involving 46 participants in total, the median age was 43–67 years and most of them did not have any formal education (Table 3). In total, eighty in-depth interviews were conducted: three CHW supervisors, three district medical officers, and two Gynaecologists-Obstetricians (Table 2).

**Table 3:** Focus group participant’s demographic information

Nr	Group	Region	# of Participants	Age (Median)	Marital status	Occupation	Schooling level
1	Matrons	Calanga	9	67	Married (3) Widow (4) Divorced (2)	Farmer (9)	Primary (9)
2		Ilha Josina Machel	6	55	Married (3) Widow (3)	Farmer (6)	Never studied (5) Primary (1)
3		Três de Fevereiro	12	65	Married (4) Widow (7) Divorced (1)	Farmer (12)	Never studied (9) Primary (3)
4		Messano	10	43	Married (5) Widow (3) Single (2)	Farmer (8) Teacher (1) Housewife (1)	Never studied (1) Primary (7) Secondary (2)
5		Chongoene	9	58	Married (2) Widow (1) Single (6)	Housewife (9)	Never studied (7) Primary (2)

### The role of community health workers in maternal care

CHW policy documents emphasise maternal and child health care. Although there is no indication on the number of visits per pregnant women policy documents state that CHWs should conduct regular home visits during pregnancy and postpartum (up to 42 days post-partum).

During these visits, CHWs interaction with pregnant women should focus mostly on health promotion, education and verification of antenatal care (ANC) attendance and to promote hospital-based delivery. The CHW training manual has a section dedicated to the care of pregnant women, with specific attention to pregnancy identification, the importance of early and regular ANC, and safe practices during pregnancy, birth preparedness counselling, the importance of delivering at the health facility and the identification of warning signs during pregnancy and delivery. Ninety-five percent of surveyed CHW reported that they are able to identify pregnant women in the community and 93 % said that they monitor pregnant women on a regular basis (see Table 4).

**Table 4:** Community health workers’ questionnaire results

	Agree	Neither Agree nor Disagree	Disagree	Unknown
Identify pregnant women	77 (95 %)	0 (0 %)	3 (4 %)	1 (1 %)
Monitor regularly pregnant women	75 (93 %)	2 (2 %)	2 (2 %)	2 (3 %)
Know the warning signs in pregnancy	75 (93 %)	0 (0 %)	3 (4 %)	3 (3 %)
Know the warning signs of hypertension during pregnancy	33 (41 %)	0 (0 %)	32 (40 %)	16 (19 %)
Know the warning signs of convulsions in pregnancy	57 (70 %)	2 (3 %)	18 (22 %)	4 (5 %)
Can identify haemorrhage during pregnancy	56 (69 %)	0 (0 %)	18 (22 %)	7 (9 %)
Can identify warning signs during labour	33 (41 %)	1 (1 %)	31 (38 %)	16 (20 %)
Can measure blood pressure	8 (10 %)	0 (0 %)	45 (56 %)	28 (34 %)
Can measure proteinuria	8 (10 %)	4 (5 %)	42 (52 %)	27 (33 %)
Can provide oral medication	38 (47 %)	0 (0 %)	25 (31 %)	18 (22 %)
Can provide oral antihypertensives	11 (14 %)	1 (1 %)	39 (48 %)	30 (37 %)
Can give injections to pregnant women	4 (5 %)	2 (2 %)	47 (58 %)	28 (35 %)
I receive additional training regarding identification and referral of pregnant women with complications	50 (62 %)	0 (0 %)	24 (30 %)	7 (8 %)
I receive regular training to identify complications in pregnancy	49 (60 %)	0 (0 %)	25 (31 %)	7 (9 %)

According to focus group discussions, matrons reported to provide advice to women throughout pregnancy, particularly related to traditional practices. The matrons do not formally offer health services, but when there is an emergency in the community they might be called upon for assistance.

Supervisors and district medical officers, hold perceptions regarding the role of CHWs which are in accordance with the existing policy documents, as illustrated in the quote bellow.

*“What they [CHWs] do when they find a pregnant woman is to refer the health facility for antenatal care, to raise awareness of the importance of antenatal consultation [and] giving birth at the health facility. It is what they do, and they issue a referral slip […] to health facility. They always make regular visits until […] after childbirth also [they] have to make follow-up to see if the child up to five years for example has completed the vaccinations [programme]. They always have to make regular visits to that family”.*- **CHWs supervisor, IDI, Gaza**

### Community health worker knowledge of the warning signs in pregnancy

CHWs training covers the identification of pregnancy warning signs, such as vaginal bleeding, fever, swelling, convulsions, severe headache, lower abdominal pain, absence of fetal movements, and weight loss.

More than half of the CHWs reported having received regular training regarding the identification of complications in pregnancy (60 %) and most (93 %) agreed that they knew the warning signs in pregnancy. Yet, less than half (38 %) of them reported not being able to identify warning signs during delivery (see Table 4). Concerning to age, older CHWs had slightly higher chance of knowing at least one warning sign in pregnancy (OR = 1.14; 95 % CI: 1.02-1.34), with relative increase associated with each year of experience or practice. However, no statistically significant association was observed for gender and education level (see Table 5).

**Table 5:** Self-reported ability of CHWs to recognise warning signs in pregnancy, pregnancy related HTA, and convulsions in pregnancy

	Total	Recognise pregnancy warning signs^b^		Recognise pregnancy hypertension warning signs		Recognise convulsions	
	N (%)	N (%)	OR (95 % CI)^a^	N (%)	OR (95 % CI)^a^	N (%)	OR (95 % CI)^a^
Total	81 (100.0)	75 (92.6)		33 (40.7)		57 (70.4)	
Sex							
Male	20 (24.7)	19 (95.0)	1.00	13 (65.0)	1.00	18 (90.0)	1.00
Female	53 (65.4)	50 (94.3)	0.88 (0.02 - 11.74)	18 (34.0)	0.28 (0.08 - 0.92)*	34 (64.2)	0.20 (0.02 - 1.00)
Missing	8 (9.9)	6 (75.0)	0.17 (0.00 - 3.82)	2 (25.0)	0.19 (0.02 - 1.44)	5 (62.5)	0.20 (0.01 - 2.24)
Age							
Median (IQR)	46.0 (35.5 - 50.0)	-	1.14 (1.02 - 1.34)*	-	0.99 (0.95 - 1.03)	-	0.99 (0.95 - 1.03)
Education							
Primary	54 (66.7)	52 (96.3)	1.00	22 (40.7)	1.00	38 (70.4)	1.00
Secondary	15 (18.5)	14 (93.3)	0.54 (0.03 - 34.01)	7 (46.7)	1.27 (0.34 - 4.68)	11 (73.3)	1.16 (0.28 - 5.73)
Missing	12 (14.8)	9 (75.0)	0.12 (0.01 - 1.21)	4 (33.3)	0.73 (0.14 - 3.15)	8 (66.7)	0.84 (0.19 - 4.39)
Years working as community health worker							
Median (IQR)	2 (1–8)	-	1.19 (0.93 - 1.94)	-	1.04 (0.97 - 1.12)	-	1.09 (0.99 - 1.24)

^a^Computed from exact logistic regression for predict upper 3 levels among the original 5 levels ordinal variable

^b^Recognised at least one warning sign

*Overall *p*-value < 0.05

Regarding haemorrhage, 69 % of CHWs who responded to the questionnaire, reported to be able to identify haemorrhage during pregnancy. Related to pre-eclampsia, only 41 % of CHWs reported to know at least one warning sign of hypertension in pregnancy (see Table 4). Female CHWs were less likely (OR = 0.28; 95 % CI: 0.08 - 0.92) to have knowledge on the warning signs of hypertension in pregnancy compared to male CHWs. This difference was not observed when comparing age, education level and years of work as CHW (see Table 5).

Seventy percent of CHWs believed they could recognise the signs of convulsions. No significant differences were observed according to the demographic variables of interest. Regarding skills to detect pre-eclampsia and eclampsia, only 10 % of CHWs reported having the capacity to measure blood pressure and proteinuria (see Table 4).

Despite not having formal training and their marginal role in the care of pregnant women in rural communities, matrons reported having knowledge of pregnancy, its complications and were able to identify warning signs such as unconsciousness, short of breath, weakness, fever, and headache. Regarding pre-eclampsia and eclampsia, matrons were unable to recognise these biomedical terms, but could list several symptoms related to these conditions including high blood pressure, convulsions and loss of consciousness. Matrons were unable to establish a direct association between specific warning signs and maternal death, except for fainting. In such cased, they perceived that the patient dies, not because the immediate physiological implications of convulsions or loss of conscience.

### Community health worker management and referral practices for pregnancy complications

Sixty-two percent of CHWs reported having received additional training regarding identification and referral of pregnant women with complications as part of regular continuous education programme. Nearly half of the CHWs reported being confident in providing oral medication of any kind (47 %), but a much lower proportion (14 %) reported confidence in specifically providing oral antihypertensives. Very few CHWs (5 %) felt confident in administering injections of any kind (see Table 4).

CHWs supervisors, district medical officers and Gynaecologists-Obstetricians showed support for task-shifting to CHWs regarding the identification and timely referral of cases before progression to severe pre-eclampsia and eclampsia. However, they showed strong skepticism regarding CHWs’ ability to manage the cases at community-level.

*“I think that it would not be ideal…blood pressure is not just any pathology which we can say: let’s create an algorithm and give it to them [CHWs] because blood pressure acts in different ways in each patient, thus it is a pathology that has to be managed by experienced people…people who are trained for that. However, if well trained they can measure blood pressure only if based on electronic devises, and refer the women to the nearest health facility”.*- **Chief medical officer, IDI, Maputo**

The scepticism was even stronger, when discussing the consideration of administration of injectable medication by CHWs to manage severe pre-eclampsia and eclampsia.

*Even if they are well trained [to administrate magnesium sulphate in the community], I would not support… I think that this will perhaps increase maternal deaths related to hypertensive disorders in the community…will he [CHWs] have and know how to correctly use magnesium sulphate in order to control hypotension and respiratory depression, for example?”.*- **Gynaecologists-Obstetricians, IDI, Maputo**

This lack of support was based on their perceptions regarding CHWs limited clinical knowledge and their eventually inability to manage adverse events of injectable drugs. They also showed concerned about CHWs limited literacy and numeracy levels and short duration of their initial training which does not include administration of injectable drugs. They insisted that in cases of an emergency, including convulsions, CHWs should immediately refer to the nearest health facility using the referral slip with the following information: date, name of the patient, age, community, referral facility, CHWs’ name, reported symptoms and signs, and any first aid or care provided.

Historically, matrons managed women during pregnancy and delivery. According to focus group findings, matrons reported to currently have less of a role in maternal care, since according to them, the Ministry of Health advised them not to manage emergencies and deliveries at home and encouraged women to seek antenatal care and delivery at health facilities.

*“Yes we help [women] giving birth but with the arrival of the hospital we were forbidden [by the Ministry of Health]. [Women] should go to the hospital. It is not because we cannot do [the] birth. We cannot do, our time ended up. And now we cannot get involved in these new things [new rules]. Our daughters are brought to hospital now”.* **Matrons, FGDs, Calanga**

However, matrons reported to still assist with deliveries outside facilities, particularly when they have a request in the late stages of labour. In the management of pregnancy complications, matrons typically use traditional methods. For example, in cases of abortion, matrons boil herbs in water, put them in a clay pot and seal it until the day of delivery. Similarly, when matrons encounter women with convulsions they treat them based on their traditional knowledge (exposing them to strong smells) before referring to a health facility.

## Discussion

This study was conducted to better understand the potential of CHWs, particularly in the provision of obstetric care at community level, with focus on pre-eclampsia and eclampsia. There were no previously published studies regarding the knowledge or competency of CHWs in identifying or managing HDPs in Mozambique. This analysis showed that despite the fact that CHWs had no specific training for identification, management and referral of pregnant women with pre-eclampsia and eclampsia, a considerable number of them reported that can identify some warning signs commonly occurring in pregnancy including: convulsions, headache, swelling among other signs. The finding that most CHWs agreed that they knew the warning signs in pregnancy likely results from the training that they receive on this topic in preparation to becoming CHWs. The ability of CHWs to identify warning signs in pregnancy is somewhat encouraging, not enough especially with regards to the link between the warning signs and the respective conditions, considering that identification of pregnant women with complications is included in their responsibilities. There is further need to improve their knowledge about pre-eclampsia and eclampsia, particularly raising their awareness on the link between hypertension and convulsions during pregnancy and on need of urgent referring pregnant women with pre-eclampsia and eclampsia being both life-threatening conditions.

Few studies assessed CHW knowledge and competencies specifically related to HDP. One study in Ghana, however, showed that CHWs reported a range of blood pressure thresholds in pregnancy, and these providers did not uniformly mention that hypertension in pregnancy was warning sign that needing referral [23]. Another study, in South Africa reviewed CHW knowledge, beliefs and attitudes related to hypertension in the general population, and found that CHWs were unaware of the causes, outcomes, prevention, and management of it. Moreover, they tended to believe in the use of traditional treatments for hypertension instead of evidence-based biomedical care, leading researchers to ultimately conclude that these health workers had insufficient biomedical knowledge related to hypertension [24].

Gender and age were the demographic characteristics which showed association with knowledge. The impact of age on this outcomes may be related to life cumulative experience as alone the years of practices as CHW did not shown any differences. It was surprising that male CHWs showed more knowledge in relation to warning signs of hypertension when compared to female CHWs. This is an important topic for further discussions and should be addressed while scaling up the intervention taking into account that gender issues can pose barriers to implementation of maternal care [25]. This study did not find significant differences in CHW knowledge according to the level of education or years of work as CHW. Despite this result, supervisors believe that CHWs’ education level was insufficient for the provision of treatment for pre-eclampsia or eclampsia. The ability to identify warning signs demonstrates that the training content is adequately recalled by CHWs, suggesting that when are well trained they can acquire practical knowledge and implement community-based interventions that can contribute to reduce maternal mortality.

Moreover, CHWs currently have health promotion and management responsibilities for other diseases such as malaria, diarrhoea and upper respiratory tract infections. The implementation of these activities has been successfully reported in various settings, suggesting that despite their low level of literacy and numeracy, with appropriate training and supervision they are capable of providing more differentiated health services at the community-level [26].

It was evident from questionnaire responses, that CHWs are not currently equipped to identify and manage hypertension in pregnancy. This is in accordance with the absence of these topics in their training manuals. Further studies to evaluate the impact of providing equipment and adequate training to assess blood pressure, measure proteinuria and manage pre-eclampsia and eclampsia should be promoted in Mozambique. It has been demonstrated in Nepal that with appropriate training of maternal health interventions, knowledge, competencies and skills can be substantially improved among village midwives [27].

This study has shown that CHWs were under pressure to refer pregnant women with pregnancy complications to the health facilities as recommended during their training, but their inability to identify most of the warning signs specific for pre-eclampsia and eclampsia may delay these referrals. A study of risk factors for eclampsia in Mozambique revealed that most referral cases reported no blood pressure measurements in antenatal clinics, indicating poor identification of women at risk [28].

CHWs seem to accept to expand their role to include management of pre-eclampsia and eclampsia although few showed confidence in administering injectable medication which is essential for the management of severe cases. This low self-confidence is also reflected among supervisors, medical officers, including specialists who believe that the CHWs are not prepared to identify and manage any complications raised from the administration of magnesium sulphate or other injectable drugs. This general scepticism can also be attributed to the fact that CHW training does not currently include administration of injectable drugs. This should be properly addressed for the successful expansion of programmes based on community interventions in the Mozambican context, also taking into account factors such as burden of work due to additional interventions, duration and quality of CHWs training, regular supervision and medication stock management [29]. Misconceptions amongst some in the medical community regarding the potential dangers of magnesium sulphate has contributed to the drug’s non-use [30]. Such misunderstandings may also lead to suboptimal practice, such as infrequent blood pressure and proteinuria measurement, and the use of diazepam in place of magnesium sulphate [31, 32]_._ At PHC level midwives are trained to, and therefore should be able to, identify and treat women with pre-eclampsia [33]. However, in Afghanistan, midwives did not identify the need for continued antihypertensive therapy in 34 % of cases [32]. Midwives, nurses and medical doctors alike have demonstrated poor performance on knowledge-based exams regarding pregnancy complications in Benin, Ecuador, Jamaica and Rwanda, the scores ranged from 51 % to 78 % on HDP-related questions [34]. Nurses and Auxiliary Nurse Midwives in Nepal similarly showed poor knowledge and skills, related to diagnosis, management and monitoring of severe pre-eclampsia and eclampsia [27]. Nevertheless, a study in Afghanistan has shown encouraging findings, the midwives where highly confident in the administration of magnesium sulphate (79 % were very confident, while 16 % had some reservations) [32].

Regarding evidence of the implementation of community based management of pregnant women, a study in Uganda suggests that trained CHW can safely provide injectable contraceptives [35]. A literature review by Malarcher (2010) also found consistent evidence that CHWs could provide injections safely, were comfortable with their ability to administer injections, and their clients were satisfied [36]. A study in Madagascar confirmed that clients were satisfied with services received from the CHW (including their administration of contraceptive injections) [37]. Many other countries have illustrated the importance of the work provided by CHWs and how they are highly regarded in their communities [33]. It is therefore reassuring that this is a window of opportunity to include tasks that are critical for maternal survival, such as administration of antihypertensive drugs. There is a need of further research addressing the ability to administer injectables by CHW in Mozambique.

Besides CHW, matrons are important and well recognised cadres of community-based maternal health care providers. However, the results of this study have shown that they are discouraged to assist emergencies and deliveries at home. Similar results were found in Ghana, where the traditional birth attendants (TBAs) were discouraged to undertake deliveries but to refer cases to health centres [38]. The role of matrons in pregnancy management may be reconsidered due to the shortage of health professionals in remote areas. The accuracy and effectiveness of matrons’ knowledge and competence is not well known, as most matrons do not receive formal training. Their skills are acquired on the basis of experience and usually taught by older and more experienced matrons [18, 19]. Training, supervision and provision of basic equipment and better coordination between matrons and health facilities would add value to their contribution in pregnancy care. It is increasingly recognized that TBAs or matrons may have a role to play in improving health outcomes in developing countries because of their access to communities and the relationship they share with women in local communities especially if women are unable to access skilled care due to long distance from health facilities, lack of money, lack of available transportation and poor health facility conditions [38, 39].

Efforts to include specific maternal health care interventions within the CHW package of training could contribute to a reduction in maternal morbidity and mortality.

### Strengths and limitations

CHWs have limited literacy and numeracy, therefore, it is possible that some respondents faced difficulties in understanding elements of the questionnaire. To minimize this concern, researchers were present during completion of the questionnaires, which may also have placed pressure on respondents and eventually effected their responses. The team made clear that their role was only to clarify the questions and not to interfere with, nor judge the answers. It was not possible to collect data from eight CHWs in Chibuto due to flooding’s; therefore, this group is not represented. No focus groups or interviews were conducted with CHWs to complement surveys responses. The assessment of knowledge and self-efficacy through use of likert scales is limited. This method does not allow respondents to provide context to their responses. In spite of this limitation, closed-ended questions were felt to be most appropriate given the sample size required to obtain representative and cross-country findings and budgetary constraints to conduct in-depth data collection among such numbers of CHWs. Despite these limitations, this study has many strengths. Quantitative methods obtain from a large sample size allowed a good overall representation of the region. All data was collected and analysed by local researchers with familiarity of the region and socio-cultural context. The mixed methods approach was an additional strength, as triangulation with the qualitative components enriched the quantitative results. This study provides novel findings regarding the knowledge and confidence in addressing the most pervasive pregnancy complications affecting Mozambicans today. Little literature is currently available regarding community health worker knowledge related to pre-eclampsia and eclampsia, and therefore these results provide unique insights.

## Conclusions

The results of this study illustrates that CHWs are aware of pregnancy complications, but have limited knowledge with regards to pre-eclampsia and eclampsia. There is a need to promote studies to evaluate the impact of enhancing their training to include additional content related to the identification and management of pre-eclampsia and eclampsia. As community health workers and matrons are the first point of contact for primary care, particularly in remote rural areas where other health services are non-existent or difficult to access, appropriate training would enforce their ability to identify, stabilize, and refer obstetric emergencies.

## Additional file

Additional file 1: Reviewer reports. (PDF 250 kb)

## Abbreviations

AMOG: Associação Moçambicana de Ginecologia
ANC: Antenatal care
AP: Administrative posts
APEs: *Agentes Polivalentes Elementares*
CHW: Community health worker
CISM: Manhiça health research center
CLIP: Community level interventions for pre-eclampsia
cRCT: Cluster randomized control trial
HDP: Hypertensive disorders of pregnancy
LMIC: Low and middle-income countries
TBAs: Traditional birth attendants
UBC: University of British Columbia
WHO: World health organization
