# Supplementary material for: Community health worker knowledge and management of pre-eclampsia in southern Mozambique
Source: Reprod Health. 2016 Sep 30;13(Suppl 2):105. doi: 10.1186/s12978-016-0220-2 (PMC5056526; doi:10.1186/s12978-016-0220-2)
Supplement: Additional file 1: — Reviewer reports. (PDF 250 kb) [file 12978_2016_220_MOESM1_ESM.pdf]

Referee's comments to the authors– this sheet WILL be seen by the author(s) and published with the article

Title	Community health care worker knowledge and management of pre-eclampsia in Mozambique
Author(s)	Helena Boene, Marianne Vidler, Orvalho Augusto, Moshin Sidat, Eusébio Macete, Clara Menéndez, Diane Sawchuck, Rahat Qureshi, Peter von Dadelszen, Khátia Munguambe, Esperança Sevene and the CLIP Feasibility Working Group
Referee's name	Julie Cliff

When assessing the work, please consider the following points, where applicable:

1. Is the question posed by the authors new and well defined?
2. Are the methods appropriate and well described, and are sufficient details provided to replicate the work?
3. Are the data sound and well controlled?
4. Does the manuscript adhere to the relevant standards for reporting and data deposition?
5. Are the discussion and conclusions well balanced and adequately supported by the data?
6. Do the title and abstract accurately convey what has been found?
7. Is the writing acceptable?

Please make your report as constructive and detailed as possible in your comments so that authors have the opportunity to overcome any serious deficiencies that you find and please also divide your comments into the following categories:

- Major Compulsory Revisions (which the author must respond to before a decision on publication can be reached)
- Minor Essential Revisions (such as missing labels on figures, or the wrong use of a term, which the author can be trusted to correct)
- Discretionary Revisions (which are recommendations for improvement but which the author can choose to ignore)

Where possible please supply references to substantiate your comments.

When referring to the manuscript please provide specific page and paragraph citations where appropriate.

General comments:

The stated aim of the study is to assess CHW knowledge and practices regarding pre-eclampsia. This has not previously been studied in Mozambique and is of relevance to low and middle income countries that rely on community health workers to provide basic maternal care in rural areas.

The methods are well-described, the data is sound and well-controlled, and the stated aim is fulfilled.

The title and abstract are accurate.

The paper is well-written, although some minor errors in English remain.

But in the discussion and conclusions the study goes beyond the stated aim in recommending that "efforts should be in place to provide equipment and to include training for the identification and management of hypertension" and that training should be enhanced "to include additional content related to the identification and management of pre-eclampsia and eclampsia".

Continued:
In recommending extra tasks for CHWs, barriers such as time constraints and logistics need to be taken into account. In 2014, the Mozambican Ministry of Health commissioned a study of additional tasks for CHWs, which the authors could usefully refer to: "Expansão do pacote de serviços dos APE em Moçambique – Vantagens e Implicações" – Ministério de Saúde, Direcção Nacional de Saúde Pública, 2014.

If the study is to go beyond its original aims and present policy recommendations, better information and evidence should be presented.

In addition, many references seem inappropriate or incorrectly numbered or are not included in the text. Examples are detailed below in Major Compulsory Revisions. Although they could individually be categorized as minor, the extent of the problem is major, and detracts from the paper. All references need to be thoroughly revised.

Major compulsory revisions:

Evidence that pre-eclampsia and eclampsia management is a recommended intervention for CHWs needs to be more clearly presented. In particular, the introductory text regarding pre-eclampsia treatment by non-physician providers beginning on line 95 needs to more accurately cite the references.
The Malawi report cited (Reference 14) appears to be the wrong reference, as it refers to training in hospitals.
Perhaps the correct reference is WHO reference 12, but this has only two mentions of eclampsia.
The second WHO report referred to (reference 13) does not recommend that CHWs should provide antihypertensives and $MgSO_4$. This should be clearly stated. If there are references that do recommend that CHWs treat pre-eclampsia and eclampsia, they should be included.
Page 17, line 352. This reference (28) refers to Afghanistan rather than Mozambique, according to the list of references. In any case, the problem of poor performance in antenatal clinics is not relevant to community health worker performance.

The following references need checking and/or correction:

Page 4, Reference 8. Need a more appropriate reference regarding use of CHWs in maternal and child care than the current one on screening for low vision.
Page 4, Reference 10 is on pre-eclampsia rather than primary health care.
Page 5, Reference 12 refers to a WHO document rather than national norms. Check that this is the correct reference.
Page 5, line 97. Reference number 14 before 13. Reference number 14 may be incorrectly numbered. Check.
Page 17, Reference 17 on Redcap incorrect. Check 16 as well.
References 21, 22, 23, 24 missing from the text or are incorrectly numbered.
Page 17, reference 28. Comes before references 25, 26 and 27. Appears to be wrong reference, as text refers to Afghanistan, not Mozambique.
Page 18, line 360. Reference 18 appears to be 23.
Page 18, line 362. Reference 19 appears to be 24.
Page 19, line 381. Reference 29. Check that correct reference.
Page 19, line 383. Reference 30. In the reference list, the Madagascar study is 35. Check.
Page 19, line 397. Reference 37 missing.

Minor essential revisions:

Page 6, Line 114. Include Maputo Province population. This may be an error, as Maputo City's population is stated.
Avoid repetitions in the methods. For example, the desk review is included under study design, data collection, and data management and analysis.
Page 12, Lines 244-247. These lines would be more appropriately included in the following section on Community Health Worker knowledge of the warning signs in pregnancy. The text does not correspond to Table 3. In table 3, some rows do not add up to 100%.
Page 13, paragraph beginning on line 255. In this paragraph, in lines 244-247, and Table 3, use uniform language: danger or warning signs, in pregnancy or of pregnancy, pregnancy or labour.
Page 14, line 284. The preceding paragraph gives no indication that CHWs are confident that they can manage obstetric emergencies. How can supervisors therefore be more skeptical?
Page 18, line 374. The score of 78% on HDP-related questions is not so low.

Correct many small errors in the reference list.

Discretionary revisions:
Page 5, Line 90. State the activities related to maternal care.
Page 6, Line 109. State the intervention being trialed here rather than later.
Page 12, Line 231. Make clear that participant details do not include those of the matrons.
Page 18, line 360. Update, as Mozambique CHWs are now providing injectable contraceptives (using a Uniject device)
Gender may also be a barrier to implementation of maternal care by male CHWs, and although mentioned, merits further discussion.

Referee's comments to the authors– this sheet WILL be seen by the author(s) and published with the article

Title	Community health care worker knowledge and management of pre-eclampsia in Mozambique
Author(s)	Helena Boene, Marianne Vidler, Orvalho Augusto, Moshin Sidat, Eusébio Macete, Clara Menéndez, Diane Sawchuck, Rahat Qureshi, Peter von Dadelszen, Khátia Munguambe, Esperança Sevene and the CLIP Feasibility Working Group
Referee's name	Joanna Morrison

When assessing the work, please consider the following points, where applicable:

1. Is the question posed by the authors new and well defined?
2. Are the methods appropriate and well described, and are sufficient details provided to replicate the work?
3. Are the data sound and well controlled?
4. Does the manuscript adhere to the relevant standards for reporting and data deposition?
5. Are the discussion and conclusions well balanced and adequately supported by the data?
6. Do the title and abstract accurately convey what has been found?
7. Is the writing acceptable?

Please make your report as constructive and detailed as possible in your comments so that authors have the opportunity to overcome any serious deficiencies that you find and please also divide your comments into the following categories:

- Major Compulsory Revisions (which the author must respond to before a decision on publication can be reached)
- Minor Essential Revisions (such as missing labels on figures, or the wrong use of a term, which the author can be trusted to correct)
- Discretionary Revisions (which are recommendations for improvement but which the author can choose to ignore)

Where possible please supply references to substantiate your comments.

When referring to the manuscript please provide specific page and paragraph citations where appropriate.

Major compulsory revisions:
Please see below.

(continue on the next sheet)

Review for Reproductive Health 7.04.16

1. **Is the question posed by the authors new and well defined?**
This study is a welcome addition to the literature, as there is very little been written about Mozambique, and it is important to understand the potential to intervene to improve maternal newborn and child health, particularly with pre-eclampsia. The question is well defined and pertinent with current recommendations to task-shift service provision to community health workers in particular.
2. Are the methods appropriate and well described, and are sufficient details provided to replicate the work?
There is not enough detail in the methods. Please see my specific comments below.
3. Are the data sound and well controlled?
I am unsure. Please see specific comments below.
4. Does the manuscript adhere to the relevant standards for reporting and data deposition?
More detail is needed.
5. Are the discussion and conclusions well balanced and adequately supported by the data?
The discussion section is interesting and well referenced, but the author is unconvinced that the arguments come from the data itself. This could be addressed by referring to my specific comments below.
6. Do the title and abstract accurately convey what has been found?
The title 'community health care workers' is abbreviated to CHWs, perhaps they would be better called community health workers as is common in the international literature? Otherwise the title and abstract reflect the findings.
7. Is the writing acceptable?
There are many typos and grammatical errors. I have pointed out several, but not all.

Abstract
First four lines - It's not clear how stating that maternal health has improved is related to the high numbers of deaths from eclampsia.
Grammar editing – self *administered* questionnaires, translated from *the* local language, *imported* into Nvivo.
Use of the word claimed indicates that you don't believe them. Reported is a better word.
Oral *medication*

Main text
Line 69 The country still *has* a – the country or women?
Line 77 it would be helpful you compared the number of doctors with WHO guidance or other countries in the region.
Line 81 could be written better.
Line 88 serve or work?
Line 88 and 89 it is not clear what the % relate to
Line 102 which study?
Line 105 it would be good if you could say why you are assessing knowledge - it feels like the sentence isn't finished.
Line 124 the research can't undertake something
Reference 16 is not properly formatted – this is important as the whole methods are described in more detail in this reference. In general the reference list has typos and is badly formatted.
Line 130 this sentence is not helpful if it is stands alone - you should discuss the types of respondents in detail so that the study can be replicated.
The structure would be improved by writing about the literature review and the data collection separately.
Were the interviews in-depth or semi structured?
Why did you sample women who are matrons? Why did you sample gynaecologists?
How many focus groups and interviews were there and where – in each study area? How many participants were there? How did you decide who to include or exclude?
Line 150 to 152 is repetitive
Was your tool based on any standard tools? International or national standards? If not, why not?
Line 160 – a likert scale is not really the best method to use while asking for attitudes
How did you analyse and use the data from the open-ended question?
Line 166 it is not clear why you did FGDs with matrons?
Line 175, it's important to know who collected data, and their skills, and this should be described in detail so that someone could replicate the study if they wished.
The discussion of the literature review is repeated several times, and this could be consolidated
Line 189 – what proportion of the data?
You can't guarantee accuracy through double entry, particularly if the data is not good at source, what was the checking process at source? How did you deal with discrepancies?
Line 189 what are cross tables?
What kind of statistical analysis?
You should have detail on the informed consent process – verbal, written etc – it is written later, but should not be discussed twice, there could be consolidation in places like this.

Line 193 and 194 – which language were the data analysed in? This is not clear
Why were the data analysed at UBC?
You should consolidate the description of data analysis of focus groups and interviews.
There is no detail on who analysed the interviews.
There is lack of detail on how qualitative data was analysed – what process or method was used, was the same coding structure used on the fgds and interviews? How were difference between UBC and the other analysing institution looked at and dealt with? The detail of this is important to report on, and would be helpful for readers doing collaborative research. Did you have skype calls to discuss the data? At what stage were themes reviewed?
Figure 2 is not labeled.
Line 202 The coding structure was developed in advance but on what basis?
Line 211 confidentiality or anonymity?
Line 215 this information is not a result of the study and should come in the introduction section
Line 219 – do you mean recruited into the study, or recruited to be CHWs? Does this include your qualitative sample as well?
Line 228 if the matrons didn't have any formal education how did this affect the written consent process?

Line 233 clumsily written
Line 247 – what kind of danger signs?
Line 259 – preeclampsia is a danger sign in pregnancy, and 93% reported knowing this, yet only 41% know about preeclampsia, you could consolidate here. In the discussion section, I tend to disagree with your argument that this is encouraging. A programme would be aiming for more awareness I think, even a national training programme?

Line 275 – it is not unusual for people to recognise that having a convulsion is a problem, it is significant to report if they do or do not recognise it as a problem in connection with the pregnancy and recognise that it is something serious that requires referral. Why do you tell us the name of convulsions in the local language? It is interesting if there is a local term for all the symptoms that come together as preeclampsia, or particularly pregnancy related convulsions, but this is just convulsions. There is a local word for convulsions in every language probably.
Line283 antihypertensive medicine is a type of medicine.
Line 284 to 294 could be condensed – both types of respondents think the same thing.

Table three. This suggest that you gave them a questionnaire which said: "can you identify the signs of haemorrhage?" and then asked them to agree (in varying degrees). This is very confusing. It doesn't make sense to ask: do you agree that you can identify the signs of haemorrhage? And you probably didn't ask that, so it is important that you give some examples of how the questions were phrased, and report the indicators more clearly in the table. For example did you collate several answers to come to the conclusions that you put in the table? If you asked them 'can you' this is really leading.

It's difficult to measure morbidities, and so it would be helpful (and is necessary for good reporting of methods) if you described how you described them and translated the tools, or maybe the tools were developed in the local language and then translated to English?
Line 299 typo

I am unsure of your rationale for considering difference of knowledge by gender and age. Are there indications from the literature that you would expect there to be differences there? It's difficult to intervene at a policy level if you are unable to explain any differences, so therefore it is important that these are theorised beforehand.
Table 4 results do not have a corresponding methods section (as mentioned above – what kind of statistical analysis)
Table 4 It seems odd that you would be interested in CHW ability to recognise one danger sign, and not the danger sign(s) that the article is focused on? Can you explain your rationale for this?

Please discuss why there is particular concern about the use of injectables among supervisors and doctors?
Can you discuss why supervisors are nervous about task shifting. Is it because of lack of referral facilities if things go wrong, or they fear for the safety of women, or is it because they might be feel threatened in some way or something else?

Line 391 this kind of information about matrons would have been useful to put in the introduction so that we know why you collected data from them.

Line 392 did you ask matrons about their connections to the health facility? It seems like these findings make sense but are not based on the data you collected.

Line 397 What are the problems that women face in accessing skilled care?

No quotations (evidence) are presented from the qualitative research, and the reader is unconvinced that the qualitative findings really added very much to the study. It would be helpful if you clarified what they added, and presented evidence.

It would have been better to have conducted qualitative research with CHWs to ask them about why they didn't feel confident about giving injections (for example). Then you know the correct policy response – maybe it is about not having enough clients to be able to apply knowledge, maybe it is about supervision, maybe it is about lack of drugs and equipment maybe it is linked with the misconceptions around the correct drugs that you mention in the discussions section – it would have been beneficial to hear from the CHWs about this. This should be mentioned in the limitations section.
Can you discuss whether or not there are weaknesses in the referral system, and the role that they play in access to care?

The limitations section needs to discuss the limitations of self-complete questionnaires beyond the literacy of the respondents. To assess skills it is often better to ask someone to demonstrate their skills or describe a case and ask them what they would do next, instead of asking them leading questions. It is difficult to comment fully on this though as it is not clear how questions were asked.

It would be beneficial to have some discussion about the feasibility to invest in matrons given the current environment pushing for institutional care.

The map is not high enough resolution so it can't be read.

The WHO has produced guidance http://apps.who.int/iris/bitstream/10665/44703/1/9789241548335_eng.pdf
It would be useful if you referenced and referred to it, especially in terms of how your study supports or refutes the recommendations. The use of job aids is mentioned etc.
Given the 'jury is out' reference to the literature on whether community health workers are able to provide eclampsia care, it would be useful if you point to the need for further research and feasibility studies on this issue.

Supplement Editor comments:

In methods of the abstract please omit the description of the tools used for the data and expand on the description of the population and methods.
